# Quality of life of patients with cutaneous leishmaniasis: A comparative analysis of the EQ-5D-3L and CLIQ questionnaires

**DOI:** 10.1371/journal.pone.0298988

**Published:** 2024-02-23

**Authors:** Endi Lanza Galvão, Janaína de Pina Carvalho, Tália Santana Machado de Assis, Mariana Lourenço Freire, Gláucia Cota, Sarah Nascimento Silva

**Affiliations:** 1 Núcleo de Avaliação de Tecnologias em Saúde, Instituto René Rachou, Fundação Oswaldo Cruz, Belo Horizonte, State of Minas Gerais, Brazil; 2 Departamento de Fisioterapia, Universidade Federal dos Vales do Jequitinhonha e Mucuri, Diamantina, State of Minas Gerais, Brazil; 3 Centro Federal de Educação Tecnológica de Minas Gerais, Contagem, State of Minas Gerais, Brazil; Universidade Federal da Bahia, BRAZIL

## Abstract

**Purpose:**

To evaluate the performance of the *Cutaneous Leishmaniasis Impact Questionnaire* (CLIQ) using the EuroQol-5 Dimension (EQ-5D-3L) as a reference standard (criterion validation); to evaluate the responsiveness of the instruments and estimate a cut-off point for the CLIQ to be able to discriminate between high and low impacts of cutaneous leishmaniasis on patients.

**Methods:**

Between 2020 and 2022, a longitudinal validation study was conducted at a reference centre for leishmaniasis in Brazil. The EQ-5D-3L and CLIQ questionnaires were administered before, during and after treatment for cutaneous leishmaniasis. The correlation between the instruments was assessed using Spearman’s correlation coefficient, responsiveness was assessed using the Wilcoxon test, and CLIQ cut-off points were proposed based on results of the EQ-5Q-3L, dichotomized between patients reporting no problems’ and ’some or extreme problems’.

**Results:**

There were satisfactory correlation coefficients between the two instruments before (-0.596) and during treatment (-0.551) and a low correlation between the instruments after the end of treatment (-0.389). In general, the responsiveness of the instruments was satisfactory. The CLIC scores that maximized sensitivity and specificity for recognizing impaired health status before and during treatment were 7 points and 17 points, respectively. However, at the end of treatment, based on the results for the EQ-5D-3L, the CLIC was not able to discriminate between individuals with high and low impacts of the disease.

**Conclusion:**

The CLIQ corresponds well with the EQ-5D-3L when applied before and during treatment but does not seem to be appropriate for follow-up evaluations after the end of treatment.

## Introduction

Cutaneous leishmaniasis (CL) is an infectious disease with a chronic course that is classified into mucosal and cutaneous clinical forms. The latter form is subdivided into localized cutaneous, disseminated cutaneous, diffuse cutaneous, cutaneous-mucosal and cutaneous relapse. The clinical presentation, in addition to being associated with host factors, has also been shown to be related to the Leishmania species involved, with more than 20 different existing species [1]. In view of the recognized limitations of investments in the diagnosis, treatment and control of the disease, leishmaniasis is classified as one of the most neglected diseases in the world [2]. In addition, the lack of surveillance and deficiencies in reporting systems in the most affected countries significantly limit determining the real burden of disease. In Brazil, according to estimates from the Ministry of Health, there was an annual rate of 7.7 cases per 100,000 inhabitants in 2021 [3].

Several studies have investigated the impact caused by the cutaneous form of leishmaniasis on psychological [4, 5], social [5, 6], professional [5], and financial [7] aspects of those affected, in addition to perceptions of treatment and access to health services by affected individuals [5, 8]. Furthermore, studies have demonstrated the deleterious effect of CL on the quality of life of people affected by the disease [9–11], increasing the levels of anxiety and depression even among children and adolescents [12].

The perceptions of health and disease status and quality of life of these patients have been assessed using two types of instruments: generic questionnaires, such as the *Revised Illness Perception Questionnaire (IPQ-R)* and the *World Health Organization Quality of Life-26 (WHOQOL-26)*, which assess the general health status of the respondent, mainly their physical and mental state [13], and questionnaires specific to skin diseases, such as the *Dermatology Life Quality Index Questionnaire (DLQI)* [13, 14], *Family Dermatology Life Quality Index (FDLQI)* [15], and *Psoriasis Life Stress Inventory (PLSI)* [13].

Recently, a specific questionnaire to assess the social, physical, occupational, economic and emotional impacts of CL was developed and validated for the Brazilian population [15]. The items on the *Cutaneous Leishmaniasis Impact Questionnaire (CLIQ)* were developed based on the clinical presentation and social context of the disease; as such, in theory, the CLIQ is the most appropriate instrument to measure the impact of the cutaneous form of leishmaniasis because it considers social, physical, occupational, economic and emotional aspects. In addition to its scope, the CLIQ provides a numerical result that represents the impact of the disease perceived by patients, allowing, for example, a comparison of data between health services and between patients receiving different treatments [15]. However, as this questionnaire was not compared to any other in its development and validation process, the criterion validity of the CLIQ, i.e., the relationship between the scores of this instrument and the scores of another previously validated instrument, has not been determined. For the same reason, cut-off values to discriminate between low and high impacts of the disease have not yet been established for this instrument. Another important step for the successful complete psychometric validation of this instrument is an evaluation of responsiveness, i.e., the ability of the CLIQ to detect changes in quality of life when there is a change in the individual’s health status.

Another instrument widely used to assess quality of life is the *EuroQol-5 Dimension (EQ-5D)*. It is a nondisease-specific questionnaire that evaluates general quality of life in five dimensions, i.e., mobility, self-care, usual activities, pain/discomfort, and anxiety/depression, measuring three levels of impairment (’no problems’, ’some/moderate problems’, and ’extreme’ problems) and a visual analogue scale (VAS) [16, 17]. This instrument, developed by the EuroQol Group, has been translated and validated for most languages, including for the Brazilian population [18–20]. The EQ-5D-3L is a three-level version of the EuroQol 5 Dimensions (EQ-5D) [21]. Despite being a widely used questionnaire with well-established psychometric measures [22, 23], even among patients with different skin conditions [24], its performance has never been evaluated in a population of patients with leishmaniasis.

In view of the lack of longitudinal evidence to define the psychometric properties of the recently developed CLIQ and the already widely validated performance of the 5D-3L EQ, the objective of this validation study was to evaluate the performance of the CLIQ using the 5D-3L EQ as the reference standard (criterion validation).

## Methods

### Study design

This was a prospective study on measurement properties based on interviews with individuals with CL.

### Setting and participants

The study was conducted at a leishmaniasis referral centre, Instituto René Rachou, Fundação Oswaldo Cruz, in Belo Horizonte, Minas Gerais, Brazil, from October 26, 2020 to May 31, 2022. The participants were consecutive patients aged above 12 years with a parasitologically confirmed diagnosis of CL who agreed to participate in the study. No exclusion criteria were applied for recruitment. Ethics approval for the study was granted by the Ethics Committee on Human Research of the Rene Rachou Institute, Fundação Oswaldo Cruz (CAAE protocol # 28929220.0.0000.5091; approval number 3,918,626, March 16, 2020). written informed consent was obtained from each participant prior to data collection. Informed permission was obtained from parents of participants under 18 years old and verbal consent was obtained from children prior to participation.

The sample size was estimated based on the minimum desired correlation between the two instruments (EQ-5D-3 L and CLIQ) [25, 26]. Thus, to estimate a medium correlation coefficient using an alpha of 0.05 and power of 90% and considering a minimum correlation coefficient of 0.4 (moderate correlation) [27], the estimated sample size was 62 subjects. To account for eventual loss to follow-up, approximately 25% extra participants were included.

### Variables and data measurements

Structured interviews were conducted by five trained researchers. Each participant was asked to answer a general questionnaire that collected sociodemographic information and the Brazilian version of the EQ-5D-3L and CLIQ. Information on medical treatments and clinical data was obtained from medical records.

The EQ-5D-3L provides a descriptive profile, and each health status problem is represented by a unique five-digit code based on severity in each dimension, ranging from no problem in any dimension (11111) to extreme in all dimensions (33333), enabling 243 different health states. Based on Minas Gerais (Brazil) population preference weights determined through the time trade-off approach [20], values ranging from -0.097 to 1 were attached to each of the EQ-5D-3L health states and were matched to each health status in the present study.

The CLIQ is composed of two scales (1: *CL general impacts* and *perceptions about health services and 2*: *Treatment*) with a total of 25 items scored on a scale from 0 to 4, yielding a total score that ranges from 0 to 100 points. When presenting two underlying constructs, the two scales’ scores are evaluated separately.

For both questionnaires, data were collected at three time points considering different clinical conditions: 1) before treatment, 2) during treatment (the period between the first dose to 30 days after the end of the last dose), and 3) at least 60 days after the last treatment dose. For the assessment carried out before the start of treatment, only the domain *CL general* scale of CLIQ impacts was used in the analysis, with the total score ranging from 0 (best health) to 72 (worst health).

### Statistical methods

For the descriptive analysis, measures of central tendency and dispersion were calculated for the quantitative variables, and absolute and relative frequencies were calculated for the categorical variables.

Statistical hypothesis tests were analysed with non-parametric tests once data were not normally distributed. The relationships between the CLIQ and the values derived from the EQ-5D-3L at the three time points were examined using Spearman’s correlation coefficient. Coefficients <0.3 were considered weak correlations, 0.30–0.50 were considered moderate correlations, and >0.50 were considered strong correlations [28]. Friedman’s test for nonparametric repeated measures was used to compare the values as well as the values of the impact of the disease measured by CLIQ at the three evaluation time points. Additional analyses were performed to assess the effect of comorbidities on the results of CLIQ. A p value <0.05 was considered statistically significant for all analyses. Additionally, to evaluate the stability of CLIQ responses over time, the responses were compared using a 2-way mixed-effect model to calculate the intraclass correlation coefficients (ICC). These methods were used to investigate the criterion validity of the CLIQ, i.e., its degree of efficacy in predicting the health status of individuals, taking into account the scores obtained with the EQ-5D-3L, which is considered the gold standard instrument for such measurements.

Responsiveness, a measure of whether an expected improvement or deterioration over a period of time is reflected in instrument scores, was tested by comparing groups based on the following question asked before beginning treatment and after the end of treatment:

Question: In general, how do you rate your health status?
Response options: very good, good, moderate, poor or very poor.

The responses were compared at the two time points evaluated and categorized as ’improvement in health status’ or ’worsening or maintenance of health status’.

Wilcoxon tests, accompanied by effect size (ES) and standard response mean (SRM) calculations, were performed to identify significant changes in the CLIQ and EQ-5D-3L scores within each category. The SRM was used to indicate how responsive the questionnaires were to changes, in which higher values would be expected for patients who rated their health status as better at the end of treatment compared to that at baseline. SRM values of 0.2, 0.5, and 0.8 represent thresholds for small, moderate and large changes, respectively [29, 30].

To determine the cut-off points for the CLIQ to discriminate between high and low impacts of the disease on patients, the responses to the EQ-5Q-3L dimensions were dichotomized into patients reporting no problems (full health status: 11111) versus patients reporting some or extreme problems (any other health status); it was considered the gold standard to guide the ROC curve analysis. Given equal weighting in sensitivity and specificity, the cut-off points were determined considering the optimized sensitivity and specificity values.

All analyses were performed using SPSS version 22. All data is organized and structured to being findable, accessible, interoperable, and reusable. It is publicly available and can be downloaded from the Zenodo data repository (https://zenodo.org/records/10214556).

## Results

A total of 78 confirmed CL patients, comprising 58 men (74.4%) and 20 women (25.6%) between 15 and 85 years of age (mean 50.5 years ± 17.3), responded to interviews conducted before the start of treatment (baseline) and were included in the study. Of these participants, 68 (87.2%) had the localized form of CL, 5 patients each (12.8%) had the mucocutaneous and disseminated forms. Three patients did not return to the outpatient clinic to start treatment, and 28 were lost to follow-up after the end of treatment. Table 1 shows the characteristics of the participants included in the study at baseline, that is, after confirmation of the diagnosis of CL, before treatment.

**Table 1:** Demographic characteristics of the patients before CL treatment.

Variables	N = 78
**Age (years), mean ± SD**	50.5 (17.3)
**Sex (n.%)**	
Male	58 (74.4)
Female	20 (25.6)
**Highest education level completed** *	
Primary school or lower	33 (42.3)
High school	30 (38.5)
College or higher	9 (11.6)
**Clinical form**	
Localized cutaneous leishmaniasis	68 (87.2)
Mucocutaneous leishmaniasis	5 (6.4)
Disseminated leishmaniasis	5 (6.4)
Number of lesions*	
One lesion	52 (66.7)
Two lesions	9 (11.5)
Three to six lesions	11 (14.2)
More than six lesions	5 (6.5)
**Presence of secondary infection**	5 (6.4)
**First CL episode**	76 (97.4)
**Presence of comorbidities**	46 (59.0)

*variable with missing information, the sum of events does not add up to 100%.

The analysis of the EQ-5D-3L results showed mean utility scores before, during and after treatment estimated at 0.786 (± 0.153), 0.823 (±0.159) and 0.846 (±0.152), respectively, with a significant difference between before and after treatment (p = 0.002); for the CLIQ, the means were 20.56 (±13.62), 22.19 (±14.64) and 14.28 (±15.88) at the respective time points, with no difference in the scores before and during treatment (p = 0.120) but between the measures performed before and after treatment (p = 0.007). In the same sense, the ICC between scores before and during treatment was high (ICC: 0.827; CI95% 0.728 to 0.890), indicating a stable response of the CLIQ. The comparison between before and after treatment had moderate reliability (ICC: 0.603; CI95% 0.354 to 0.756) while the reliability during and after treatment was 0.697 (CI95% 0.502 to 0.812).

When evaluating the influence of comorbidities in assessing the impact of leishmaniasis on health-related quality of life, it was observed that the CLIQ score did not differ significantly between patients with and without comorbidities at different assessment times. There was no significant difference in the CLIQ scores before and during the treatment for patients with and without comorbidities (p = 0.165 and p = 0.161, respectively). But this subgroup analysis showed differences in CLIQ scores before and after treatment (p = 0.023 and p = 0.004, respectively) and during and after treatment (p = 0.023 and p < 0.001, respectively).

The correlations between the values of these two instruments for determining the criterion validity of the CLIQ, i.e., its ability to predict the health status of individuals, considering the EQ-5D-3L as the reference standard, at baseline (before the beginning of treatment), during treatment and after the end of treatment are provided in Table 2.

**Table 2:** Criterion validity using Spearman’s rank correlation coefficient.

	EQ-5D-5L (utility)		
	Before the start of treatment (n = 78)	During treatment (n = 75)	After the end of treatment (n = 47)
**CLIQ**	- 0.596 ^a^ P < 0.001	- 0.551 ^a^ P < 0.001	- 0.389 ^a^ P = 0.007

^a^ Pairwise correlation coefficients in the expected direction.

Regarding the comparison of health status before and during treatment, four patients reported worsening, 25 perceived improvements, and 18 did not perceive changes. Thirty-one patients did not participate in the interview during treatment. Considering only patients who reported maintenance or worsening of their general health status, a significant change was observed in EQ-5D-3L scores (p = 0.009) but not in CLIQ scores (p = 0.755) when comparing the baseline scores with the end of treatment scores. Among patients who reported improvements in their general health status, there was a significant change in CLIQ scores at these evaluation times (p = 0.006) (Table 3).

**Table 3:** Responsiveness of the CLIQ and EQ-5D-3L.

Items	*No*.	Before treatment (mean)	After the end of treatment (mean)	Mean change ^a^	SD at baseline	SD at change	ES	MRS ^b^	*p-* value
**EQ-5D-3L**									
Improved health status	25	0.762	0.829	0.066	0.178	0.156	0.373	0.424	0.056
Maintenance or worsening of health status	22	0.794	0.869	0.074	0.160	0.113	0.463	0.654	0.009*
**CLIQ**									
Improved health status	25	23.56	14.73	-8.82	13.28	-8.820	0.664	0.590	0.006*
Maintenance or worsening of health status	22	19.55	20.59	1.04	14.16	12.843	0.073	0.081	0.755

^a^ Mean change: *posttreatment–before treatment*

^b^ SRM, standardized response mean: *(mean baseline score − mean follow-up score)/(SD of change score)*

* P < 0.05

**Fig 1:**
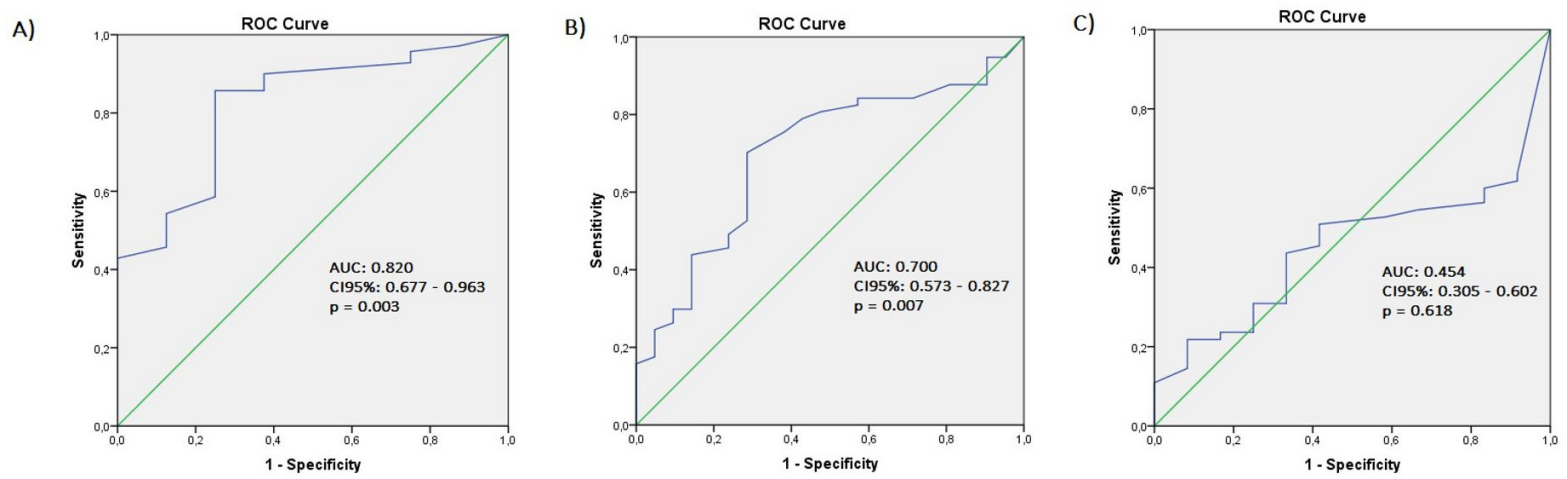
Receiver operating characteristic curves (ROC curves) for CLIQ cut off scores based on health status. (A) Before treatment, (B) During treatment, (C) After the end of treatment. AUC: area under the curve; CI: confidence interval.

Before the start of treatment, 8 patients (10.3%) rated their health status for the five dimensions of the EQ-5D-3L as ’no problems’; the remainder (89.7%) reported some limitation or problem in at least one of the following aspects: mobility, personal care, usual activities, pain or discomfort, anxiety and depression. During treatment, 21 patients (26.9%) reported a full health status, decreasing to 12 patients (15.4%) after the end of treatment. By parameterizing these results with the CLIQ scores for before, during and after treatment, ROC curves were obtained (Fig 1).

Using the health status defined by the EQ-5D-3L as a reference, the discriminatory power of the CLIQ in the prediction of no problems versus some or extreme problems was evaluated. For the time points before and during treatment, the CLIC performance was considered statistically significant (AUC: 0.820 95% CI 0.677–0.963, p = 0.003; and 0.700 95% CI 0.573–0.827, p = 0.007, respectively). However, in the interview at the end of treatment, based on the EQ-5D-3L scores, the CLIC was not able to discriminate between individuals with high and low impacts of the disease (AUC: 0.454 CI95% 0.305–0.602, p = 0.618).

The CLIC score that was related to the best accuracy for the recognition of some impairment of health status (sensitivity 75.0% and specificity 85.7%, positive and negative predictive values of 37.5% and 96.7%, respectively) before treatment was 7 points. Based on this observation, it is possible that values from 0 to 7 indicate a low impact of the disease (or the equivalent of a full health status) and that values above 7 identify patients with a high impact (or equivalent to some degree of impairment of health status). For evaluations performed during treatment, the cut-off point that maximized sensitivity (71.4%) and specificity (70.2%) was 17 points (positive and negative predictive values of 46.8% and 86.9%, respectively). As the correlation between the CLIQ and the EQ-5D-3L after the end of treatment was very low and the ROC curve was unable to discriminate between subjects who perceived high or low impacts of the disease, a cut-off score was not calculated for this time point.

## Discussion

There were some important findings in this study. This was the first study to apply the EQ-5D-3L questionnaire to investigate the health status of patients with CL. In addition, the validity of the CLIQ quality of life questionnaire was investigated in a population different form that for which the instrument was developed, using the EQ-5D-3L, an instrument with well-established psychometric properties, as a reference parameter [31–33].

We found satisfactory correlation coefficients between these instruments before and during treatment, indicating a relationship between the general health status perceived by the patients and the impact caused by the disease. The area under the curve (AUC) provides an estimate of the probability of correctly classifying a subject at random (test accuracy). In this study, the ROC curve results indicated that individuals with CL, when recognizing impairment in their health status, also perceive an impact of this disease on their quality of life; this occurred for 82% of participants before beginning treatment and for 70% of participants during treatment. Notably, the correlation was inverse because the instruments have opposite directions: for the CLIQ, higher scores indicate a greater impact of the disease and treatment, and for the EQ-5D, higher utility values are considered ideal. However, there was a low correlation between the instruments after the end of treatment, potentially indicating that factors other than CL probably influence the health status of the individuals at this time point. Although the correlation between the two instruments was significant and satisfactory, in general, the reference values for the correlation coefficients presented in the scientific literature are arbitrary and should be used with caution [34].

The CLIQ showed stable results before and during treatment, with less consistency when comparing before and after treatment, exceeding 10% between the two assessments of ICC. The course of treatment could cause fluctuations in the responses and important differences in the responses over time are expected considering any clinical change. In this study, the longitudinal follow-up allowed verifying the responsiveness of the CLIQ; responsiveness is an important measure to understand the ability of an instrument to detect changes in scores based on changes in disease progression or in the clinical status of patients [35]. In this study, the EQ-5D-3L was unable to identify a significant change in the health status of those patients who reported improvements in this condition between baseline and the end of treatment. Interestingly, this instrument identified a significant increase in utility values among those patients who reported maintenance or worsening of their health status. In contrast, good CLIQ responsiveness was identified, as the CLIQ successfully captured the positive changes that correspond to improved health status, as suggested by the ES and SRM values. According to the scientific literature, disease-specific scales, because they are more focused and adapted to problems of particular importance to a target group of patients, are generally more responsive than are generic measures of health status [36], which may explain the difference in performance between the instruments. Thus, for future studies of a longitudinal nature, the CLIQ is more appropriate.

The score defined as the cut-off point for the CLIQ varied based on the time point. In this study, a cut-off point > 7 for the CLIQ assessed before the start of treatment showed very good sensitivity to discriminate individuals with high and low impairment of quality of life. Similarly, a cut-off point > 17 for the disease and treatment impact scales applied during treatment was also able to discriminate these individuals, with good sensitivity and specificity. The literature emphasizes the importance of considering sensitivity the most important indicator to minimize the chance of false-negative cases, increasing the ability of the instrument to identify patients with true impairment [37].

Most study participants were identified as having a high perceived impact of CL. Until recently, only 1 study had performed a similar analysis using the CLIQ, suggesting a cut-off point at the median [7]. Thus, the current results are important because they allow comparability of results between different populations; notably, the cut-off point at the median makes external validity of the results unfeasible [38]. Although this questionnaire was specifically tested in research context, it can also be used as an important measurement tool in clinical practice once it is relatively quick to complete and easy to analyse.

The main limitation of the present study is that it was conducted in a single centre with a relatively small number of patients. In addition to the relatively low number of patients, the data at follow-up time after the treatment were incomplete and not all patients were always evaluated. These factors could introduce bias and impact the study’s generalizability and internal validity. Interpretation of results related to the interviews after the treatment requires caution. The sample shows a significant gender imbalance, which could limit the generalizability of the findings. In the same context, we could not analyse in depth the influence of age and their impact on the results. The small number of children with CL diminishes the power of our results, and the external validity to this population is limited. Thus, the convenience sampling may have led to selection bias. Finally, the study lacks comparative analysis with other established metrics or treatment protocols, what would provide a comprehensive understanding in the broader context of CL management.

Notably, the two instruments evaluated were developed for different uses and, therefore, capture distinct but correlated experiences. These findings are useful for understanding the association between impaired quality of life and health status in individuals with CL. Considering a drop in the correlation between CLIQ and EQ-5D-3L after treatment, CLIQ might not be the most suitable measure for long-term impact evaluation. Future studies should consider a multicentre sample to incorporate greater clinical variability to assess the responsiveness of the instruments. Once CLIQ was developed for the Brazilian population, the translation and cross-cultural validation, as well as the evaluation of its responsiveness across different cultural settings, should be aims of future studies.

## Supporting information

S1 Appendix STROBE statement—Checklist of items that should be included in reports of observational studies. (DOC)

## Abbreviations

AUC: Under the curve
CL: Cutaneous Leishmaniasis
CLIQ: Cutaneous Leishmaniasis Impact Questionnaire
DLQI: Dermatology Life Quality Index Questionnaire
EQ-5D-3L: EuroQol-5 Dimension
ES: Effect size
FDLQI: Family Dermatology Life Quality Index
ICC: Intraclass Correlation Coefficient
IPQ-R: Revised Illness Perception Questionnaire
PLSI: Psoriasis Life Stress Inventory
ROC: Receiver operating characteristic
SRM: Standard response mean
WHOQOL-26: World Health Organization Quality of Life-26
